# Multidimensional analysis explores the correlation and potential molecular mechanisms between serum uric acid and risk of gastric cancer

**DOI:** 10.3389/fmed.2026.1929597

**Published:** 2026-10-28

**Authors:** Wene Wei, Fuxing Zhao, Qi Fu, Xian Liang Shen, Hongxia Liao, Ji Di

**Affiliations:** 1 College of Clinical Medicine Qinghai University, Xining, China; 2 Department of Medical Oncology Ward two of Affiliated Hospital of Qinghai University, Affiliated Cancer Hospital of Qinghai University, Xining, China; 3 Breast Disease Diagnosis and Treatment Center of the Affiliated Hospital of Qinghai University, Affiliated Cancer Hospital of Qinghai University, Xining, China; 4 First People’s Hospital of Xining, Xining, China

**Keywords:** gastric cancer, mechanisms, multidimensional analysis, risk, serum uric acid

## Abstract

**Objective:**

To investigate the causal association between SUA and gastric cancer risk and elucidate the underlying biological mechanisms.

**Methods:**

A two-sample Mendelian randomization (MR) design was adopted. The inverse-variance weighted (IVW) method served as the primary analysis, supplemented by MR-Egger, weighted median, simple mode and weighted mode methods. Sensitivity analyses included the MR-Egger intercept test for horizontal pleiotropy, Cochran's *Q*-test for heterogeneity, and leave-one-out analysis. SNP-mapped genes were analyzed for expression profiles, prognostic value and functional enrichment in gastric cancer using TCGA data. *In vitro* validation was performed in uric acid-treated AGS gastric cancer cells to assess cell viability, apoptosis, oxidative stress markers and PI3K/AKT pathway activity.

**Results:**

Genetically elevated SUA was causally associated with reduced gastric cancer risk in the IVW analysis (OR = 0.76, 95% CI: 0.62–0.92, *p* = 0.006), with consistent directional estimates across other MR methods. Sensitivity analyses indicated significant heterogeneity (Cochran's Q, *p* = 3.5 × 10⁻⁵) but no directional horizontal pleiotropy (MR-Egger intercept, *p* = 0.185). Two outlier SNPs were identified by MR-PRESSO, and outlier-corrected estimate remains significant (OR = 0.88, 95% CI: 0.77–1.00, *p* = 0.048), which confirmed the robustness of the SUA protective effects. Among 36 identified SNP-mapped genes, 25 were differentially expressed in gastric cancer, and 6 (ABCG2, BICC1, CFAP73, GNAS, MXD3, PNPLA3) were significantly associated with overall survival. These genes were enriched in uric acid metabolism, carboxylic acid transport and the mTOR signaling pathway. *In vitro*, uric acid dose-dependently inhibited proliferation, induced late apoptosis/necrosis, increased intracellular ROS, depleted antioxidant defenses, and suppressed PI3K/AKT pathway activity in AGS cells.

**Conclusions:**

SUA exerts a protective causal effect against gastric cancer. The anti-tumor effect is mechanistically driven by oxidative stress induction and PI3K/AKT pathway inhibition, with SNP-associated genes linked to gastric cancer progression via metabolism-related signaling pathways.

## 1 Introduction

Gastric cancer is a lethal tumor that affects the digestive system and ranks as the fifth most prevalent type of cancer globally, as well as the fourth leading cause of cancer-related mortality (1). Serum uric acid (SUA) is a byproduct of the digestion of foods and beverages containing purine nucleotides, specifically, SUA is formed by the oxidative catalysis of xanthine and hypoxanthine by xanthine oxidoreductase (2). Current research suggests that SUA is associated with the onset and progression of various diseases including cancer (3–6).

Current research has demonstrated a biological correlation between SUA levels and cancer incidence of cancer (7). The mechanisms by which SUA contributes to the pathogenesis of malignant tumors are multifaceted (8–10). A meta-analysis has shown that SUA is positively correlated with the risk of liver cancer and colorectal cancer but negatively correlated with the risk of lung cancer, suggesting that the dual effects of SUA may vary depending on the type of cancer (2). Additionally, a meta-analysis of 33 independent cohort studies indicated a correlation between SUA levels and overall cancer incidence (11). More recently, a systematic review and meta-analysis reported significantly elevated SUA levels in gastric cancer patients despite considerable heterogeneity across studies (12). However, it remains unclear whether SUA serves as a protective factor or risk factor for gastric cancer; thus, the subject continues to be a matter of debate in current research.

At the molecular level, a dual role of uric acid in tumorigenesis was documented. SUA has been proposed to function as a reactive oxygen species (ROS) scavenger to protect against oxidative stress-related carcinogenesis in view of its distinct feature as the most abundant endogenous antioxidant (13). On the other hand, intracellular and high concentration of uric acid severely impair antioxidant defenses such as SOD, catalase and glutathione, which eventually triggers oxidative stress-mediated cell apoptosis (14). The intracellular redox status further modulates the PI3K/AKT pathway, which is involved in cell survival, proliferation and metabolism and frequently hyperactivated in gastric cancer. Therefore, the potential contributions of uric acid to gastric cancer through either redox homeostasis or cellular survival signaling are still to be elucidated at both genetic and cellular levels.

Currently, the association between SUA levels and the risk of gastric cancer remains unclear. One study indicated that SUA is linked to poor prognosis in gastric cancer patients, although no evidence has established its role in increasing the incidence of the disease (15). Further investigation is required to clarify the relationship between SUA and gastric cancer and elucidate the underlying mechanisms. A 2024 Mendelian randomization (MR) study found no causal relationship between SUA and the pathogenesis of gastric cancer (16). In contrast, another study reported a nonlinear association, with gastric cancer incidence initially decreasing and then increasing as SUA levels rise (17). Thus, existing findings on the correlation between SUA levels and the risk of gastric cancer are inconsistent, underscoring the need for additional research to further explore this relationship.

Existing research has many confounding factors and may not accurately reflect the true relationship between SUA levels and gastric cancer incidence. MR can eliminate confounding variables and more accurately reflect the relationship between SUA levels and the risk of gastric cancer. MR is currently an important epidemiological tool used to investigate possible causal relationships between exposure and outcomes. Using genetic variation as an instrumental variable can more accurately describe the causal relationships between variables, help alleviate the influence of confounding variables, and enhance the robustness of causal inference (18, 19).

The aim of this study was to analyze the correlation between SUA and the risk of gastric cancer through MR and to use bioinformatics methods to analyze the biological functions of genes related to both SUA and gastric cancer in gastric cancer and their impact on gastric cancer prognosis. In addition, gastric cancer cells were cultured in a medium containing uric acid to further explore the effect of uric acid on the biological functions of gastric cancer cells, thereby exploring the effect of uric acid on the pathogenesis of gastric cancer and its potential mechanisms.

## 2 Methods

### 2.1 Mendelian randomization

#### 2.1.1 MR design

We conducted a Mendelian randomization analysis using SUA as the exposure factor and gastric cancer as the outcome measure to assess the potential causal relationship between SUA and the risk of gastric cancer. To ensure the validity of the instrumental variables, the following three core assumptions must be satisfied: (1) the instrumental variables are strongly associated with the exposure measure (SUA), (2) the instrumental variables are not associated with any potential confounders, and (3) the instrumental variables influence the outcome only through the exposure, meaning that they have no direct association with gastric cancer. Initially, single nucleotide polymorphisms (SNPs) significantly associated with SUA were screened based on genome-wide association study data, with a significance threshold set at *p* < 5 × 10⁻^8^. To eliminate the effects of linkage disequilibrium, SNPs were pruned using PLINK in the TwoSampleMR package, with a *r*^2^ threshold of 0.001 and a 10,000-kb window, based on the 1,000 Genomics Project European reference panel. Subsequently, through manual verification, SNPs associated with cancer outcomes (*p* < 5 × 10⁻^5^) or with known confounding factors were excluded. Finally, the number of selected instrumental variable SNPs should be moderate, typically ranging from several dozens to a few hundred, to ensure the validity and statistical power of the analysis. The data harmonization and the primary MR analysis was performed in R (4.6.1) with TwoSampleMR (0.7.9) (20). Five MR estimators were employed in this study including: inverse variance weighted (IVW) (21, 22), MR-Egger regression (23), weighted median estimator (24) and simple and weighted mode estimator (25). MR-PRESSO (1.0) (26) global test, outlier test and distortion test were adopted for sensitivity analysis. The R scripts are available upon request.

#### 2.1.2 Data sources

We obtained information from the IEU Open GWAS database (https://gwas.mrcieu.ac.uk, Accessed on March 15, 2023) query and download SNPs data on gastric cancer and SUA.

#### 2.1.3 MR analyses

Read GWAS data of exposure factors (SUA) and select appropriate tools, usually set to *p* < 5 × 10^−8^, and if necessary, perform clumping. Read the GWAS data of the outcome variable (gastric cancer) and extract the SNPs (single-nucleotide variations with a mutation frequency greater than 1%) of the instrumental variables mentioned above. Data preprocessing: GWAS data on exposure factors and outcomes were preprocessed to standardize their format. We used the default MR function to use five algorithms for MR analysis (MR Egger, Weighted media, IVW, Simple mode, weighted mode). IVW is mainly used for MR analysis, supplemented by MR Egger regression, the weighted mode method (WM), and the weighted median method (WME). Visualization of analysis results: Scatter plots, forest plots, and funnel plots, where the slope of the scatter plot represents the causal effect of exposure factors on outcomes.

#### 2.1.4 Horizontal pleiotropy and heterogeneity test

We applied the MR-Egger regression technique to estimate pleiotropy and assessed heterogeneity using Cochran's *Q*-test. To mitigate the potential for horizontal pleiotropy, the significance of the MR-Egger intercept was verified to be greater than 0.05. If the *p*-value from Cochran's *Q*-test was less than 0.05, we resorted to the random-effects IVW model as our final outcome; otherwise, we employed the fixed-effects model. The F-statistic was used to gauge the strength of the association between the SNPs and exposure. An F-statistic exceeding 10 indicated the absence of a weak IV. A comprehensive statistical analysis yielded statistically significant differences (*p* < 0.05).

#### 2.1.5 Leave-one-out analysis

Each SNP was gradually removed, the meta-effect of the remaining SNPs was calculated, and the results were observed after removing each SNP. If the result changes significantly after removing a certain SNP, it indicates that there is a significant effect on the result. Ideally, the results should not change significantly after gradual removal of a certain SNP.

### 2.2 Bioinformatics analysis

#### 2.2.1 Screening of SNPs corresponding genes

We screened the genes corresponding to the SNPs included in the Mendelian randomization forest plot and analyzed their expression, prognosis, and biological functions in gastric cancer. We used the NCBI database to identify genes corresponding to specific SNPs using the following steps: (1) Visit the NCBI website https://www.ncbi.nlm.nih.gov. (2) The dropdown menu in the top search bar was used to select the “SNP” database. (3) Enter the SNP ID (rsID) in the search bar and click “Search.” (4) On the SNP summary page, locate the section marked as “gene” or “mapped gene” to find the gene corresponding to the SNP.

#### 2.2.2 Data acquisition

Based on the genes corresponding to SNPs found in the NCBI database, we selected gastric cancer RNA-seq data from TCGA database for analysis, including differential expression of these genes in gastric cancer, prognostic analysis, and biological function analysis.

#### 2.2.3 Differential gene expression analysis

We conducted differential gene expression analysis to compare gastric cancer tissues with adjacent normal tissues. This analysis employed the “edge” R package, which is specifically tailored for RNA-seq data differential expression studies. Genes were classified as differentially expressed if they met the criteria of an absolute log2 fold-change (|log2FC|) greater than 1 and an adjusted *p*-value below 0.05, following Benjamini-Hochberg correction for multiple testing.

#### 2.2.4 Prognostic analysis

The “survival package” was used to conduct proportional hazards hypothesis testing and fit survival regression, and visualize the results using the “survminer package” and “ggplot2 package”. Cox regression was used for statistical analysis.

#### 2.2.5 Gene functional analysis

To elucidate the biological significance of differentially expressed genes (DEGs), we performed enrichment analyses for Gene Ontology (GO) terms and Kyoto Encyclopedia of Genes and Genomes (KEGG) pathways using the clusterProfiler R package. GO terms and KEGG pathways were considered significant if they had an adjusted *p*-value of less than 0.05.

### 2.3 Cell experiment exploring the effect of uric acid on AGS gastric cancer cells

#### 2.3.1 Cell culture and uric acid treatment

AGS cells (CRL-1739, ATCC. STR-authenticated, mycoplasma-free, RPMI-1640 supplemented with 10% FBS and 1% penicillin-streptomycin) were seeded in 96-well plates at a density of 5 × 10^4^ cells/mL (100 µL/well) and incubated for 24 h at 37 °C under 5%CO_2_. Uric acid (U2625, Sigma) stock solution was prepared in pre-warmed culture medium containing 1 mM NaOH and immediate diluted into complete culture medium before use. After attachment, the cells were treated with fresh medium containing uric acid at final concentrations of 0 μM (control), 50 μM, 100 μM, and 200 μM for 48 h, with 3 independent biologic replicates unless specified.

#### 2.3.2 Cell viability assay

Following treatment, cell viability was measured using CCK-8 kit (CK04, Dojindo). Briefly, the medium was completely aspirated, and 100 μL of the CCK-8 mixture was added to each well. The plate was incubated for 2 h at 37 °C with 5%CO₂, and the absorbance at 450 nm was measured using a microplate reader (Spectramax iD3, Molecular Devices). Viability was calculated as a percentage relative to the vehicle control.

#### 2.3.3 Apoptosis assay

After 48 h of treatment with uric acid, cells and supernatants were collected, centrifuged (500×g, 5 min), and washed with PBS. The pellet was resuspended, counted (≥10^5^ cells), and washed with 1 × Binding Buffer. Cells were stained with Annexin V-FITC (5 μL, 15 min, RT, dark) and PI (10 μL, 5 min, ice, dark) in 400 μL Binding Buffer according to the manufacturer' instruction (40302ES, Yeasen) and analyzed by flow cytometry (CytoFLEX, Beckman) within 30 min. The early apoptotic (Annexin V⁺/PI⁻) and late apoptotic/necrotic (Annexin V⁺/PI⁺) cells were quantified.

#### 2.3.4 Detection of levels of superoxide dismutase (SOD), catalase (CAT), and glutathione (GSH)

AGS gastric cancer cells were treated with uric acid, and the levels of SOD, CAT, and GSH were detected using SOD (EIASODC, ThermoFisher), CAT (EIACATC, ThermoFisher), and GSH (EIAGSHC, ThermoFisher) detection kits, respectively according to the manufacturer's instructions. Results were normalized to total protein content.

#### 2.3.5 Flow ROS detection

Cells were cultured in media containing (*n* = 3 per group) for 48 h. After cultivation, the cells were collected, washed with PBS, and incubated with 10μmol/L DCFH-DA in serum-free medium at 37 °C for 20 min. After washing three times, ROS levels were analyzed using flow cytometry. The percentage of DCF-positive cells was quantified.

#### 2.3.6 Western blot analysis of the PI3K/AKT pathway

Western blotting was used to detect the activity of key proteins in the PI3K/Akt pathway after uric acid treatment of AGS cells. First, cells were divided into a control group and groups treated with different concentrations of uric acid. Total protein was extracted, and the concentration was measured using the BCA method (23,225, ThermoFisher). After adjusting the concentration to a uniform value, the cells were denatured and set aside for later use. Next, SDS-PAGE was performed to separate equal amounts of proteins and transfer them to the PVDF membrane. After blocking non-specific sites, the membrane was incubated with primary antibodies (PI3K (1:1,000, EPR18702, Abcam), total Akt (1:5,000, EPR16798, Abcam), phosphorylated Akt (1:1,000, EPR18853, Abcam), and internal reference *β*-actin (1:5,000, EPR16769, Abcam) antibody) and secondary antibodies (1:5,000, ab6721, Abcam) in sequence. Finally, ECL (32,106, ThermoFisher) imaging was performed. During data analysis, software such as ImageJ was used to quantify the grayscale values of the bands, and the degree of Akt activation was evaluated by the ratio of phosphorylated Akt to total Akt. Statistical analysis was conducted to compare the differences between the groups and to reveal the effect of uric acid on the PI3K/Akt pathway.

## 3 Results

### 3.1 MR analyses

We obtained ebi-a-GCST90018849(includes a sample size of 476,116 and a total of 24,188,662 SNPs, from European populations) related to gastric cancer from the IEU Open GWAS database. Similarly, we obtained ebi-a-GCST90018757(including a sample size of 129,405 and 12,499,459 SNPs from East Asia) data related to SUA from the IEU Open GWAS database. Both datasets were derived from the cross-population GWAS analysis of 220 human phenotypes by Sakaue et al. (27). The results showed that IVW showed a statistically significant result with an OR of 0.76(95%CI: 0.62–0.92, *p* = 0.006). The MR Egger yielded an OR of 0.94(95% CI: 0.68–1.31, *p* = 0.73). The WME produced an OR of 0.95(95% CI: 0.81–1.09, *p* = 0.44). The Simple mode method resulted in an OR of 0.76(95% CI: 0.58–1.01, *p* = 0.06). Finally, the WM gave an OR of 0.98(95% CI: 0.87–1.10, *p* = 0.70). In summary, all methods indicated a directionally negative association, but only the IVW estimate reached the conventional levels of statistical significance (Figure 1).

**Figure 1:**
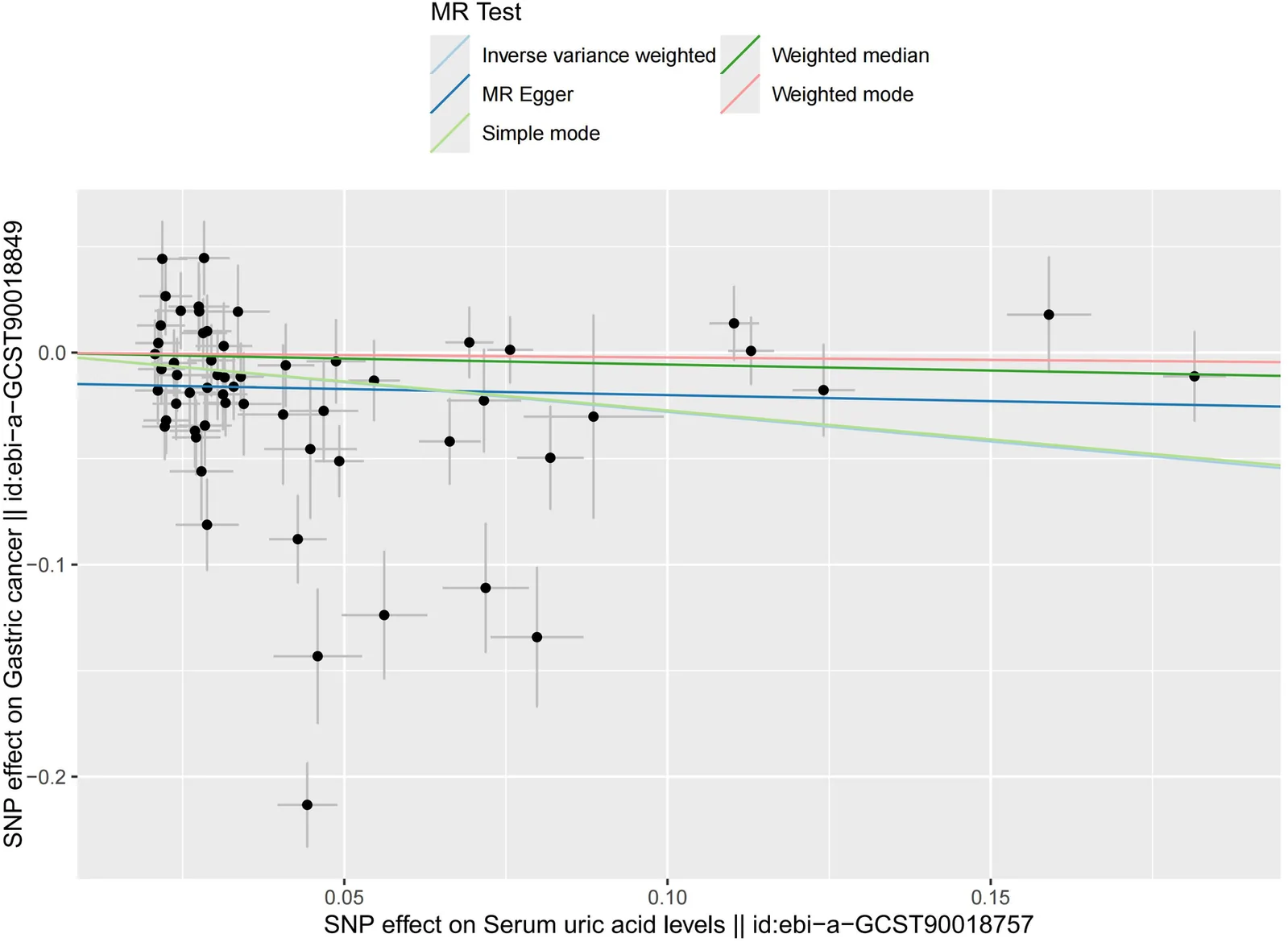
Scatter plot of MR analysis for the causal effect of SUA on gastric cancer. Each point represents one SNP, and slopes of fitted lines (IVW, MR-Egger, weighted median) indicate the association between elevated SUA and reduced gastric cancer risk (IVW OR = 0.76, 95%, CI: 0.62–0.92).

### 3.2 Horizontal pleiotropy and heterogeneity test

The funnel plot shows an asymmetric shape (Figure 2). The high-precision points are not clustered near the IVW bus line but are biased towards the MR Egger line side. This asymmetric suggests directional level pleiotropy, indicating that some instrumental variables may affect outcomes through pathways other than exposure, leading to bias in the IVW estimation results.

**Figure 2:**
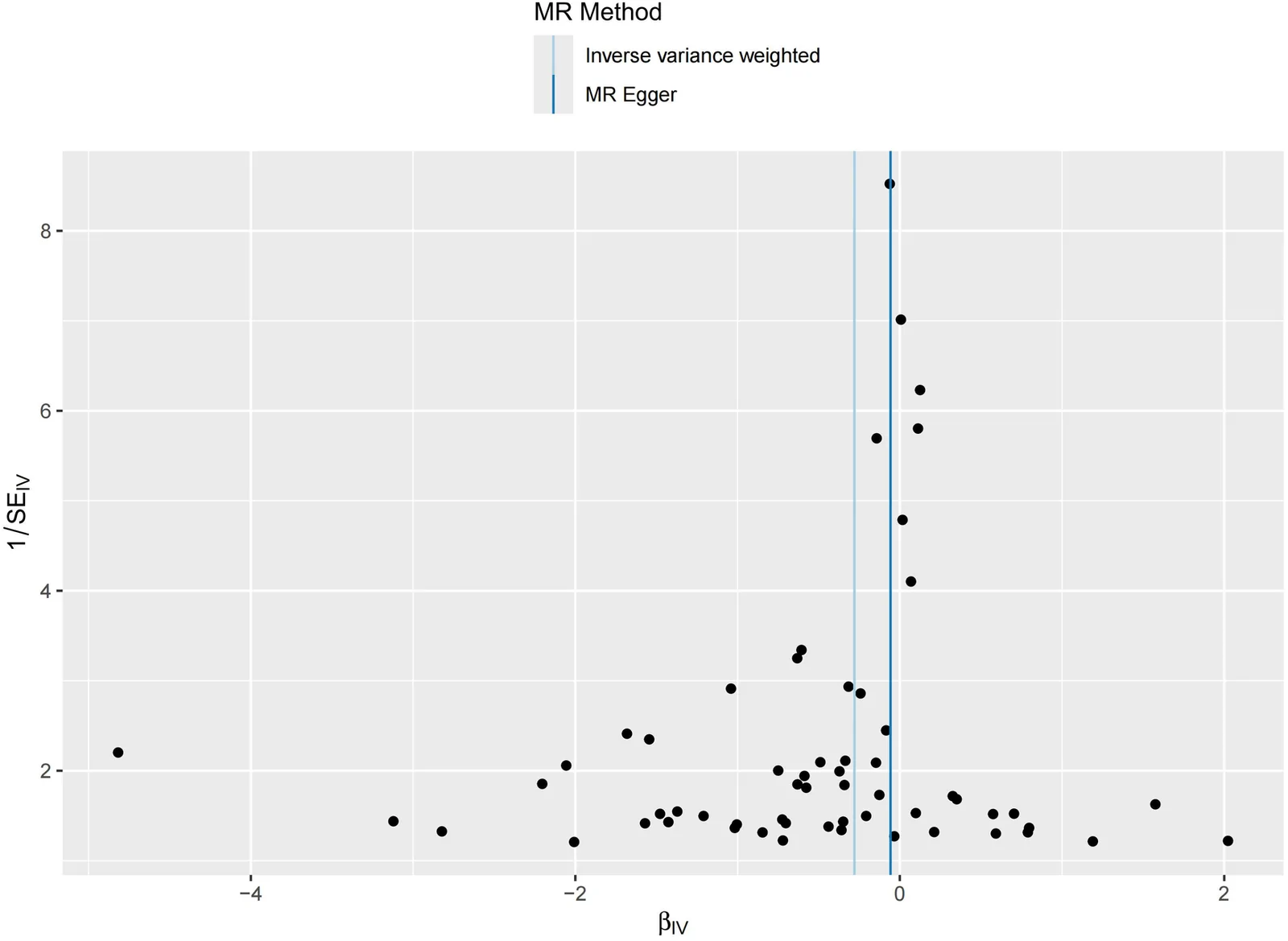
Funnel plot of MR analysis of SUA on gastric cancer risk. The vertical lines represent the causal estimates from the IVW (symmetric) and MR-Egger (asymmetric) methods. The asymmetry of the point distribution suggests potential directional horizontal pleiotropy.

### 3.3 Sensitivity analysis

All instrumental variables were strong instruments (per-SNP F-statistics: 30.00–1,409.80; overall F-statistic: 174.82). The significant heterogeneity among the SNP estimates was indicated by Cochran's *Q*-test (Q = 109.76, *p* = 3.5 × 10⁻⁵), while no evident directional horizontal pleiotropy (intercept = −0.0085, *p* = 0.185) was observed in the MR-Egger intercept test. MR-PRESSO identified two pleiotropic outlier SNPs (rs80292863, rs9468498), and distortion test indicated these outliers' removal did not significantly alter the causal estimate (*p* = 0.396). It's noteworthy that the residual pleiotropy detected by MR-PRESSO global test was consistent with the asymmetry observed in the funnel plot. Leave-one-out estimates and their confidence intervals are closely clustered together, forming a stable “band,” which indicates that the final conclusion will not essentially change regardless of which SNP is excluded (Figure 3).

**Figure 3:**
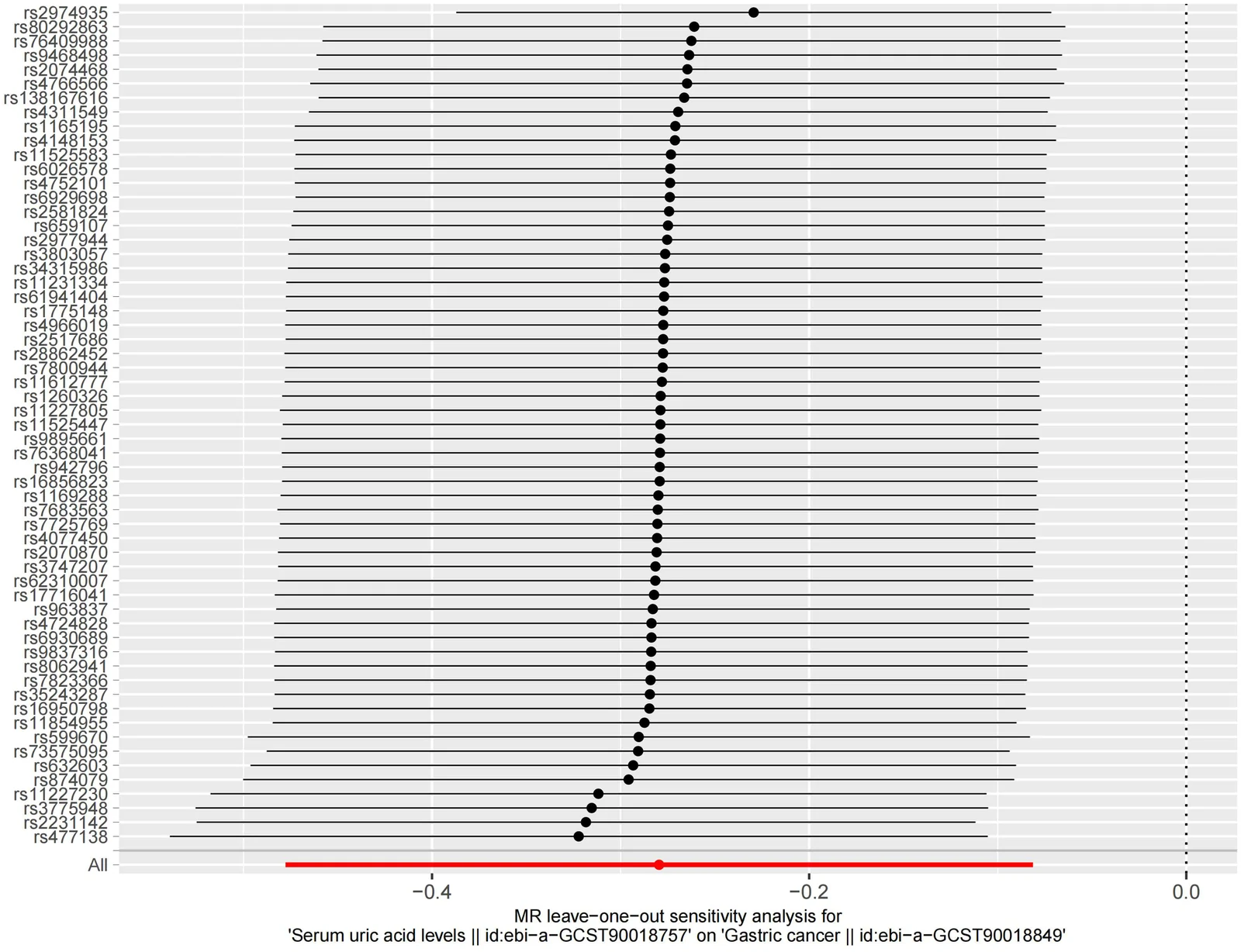
MR leave-one-out sensitivity analysis for SUA on gastric cancer risk. The forest plot represents the causal odd ratios and 95% confidence intervals after exclusion of each instrumental SNP.

### 3.4 Search for genes based on SNP

Based on Mendelian randomization analysis, 59 SNPs were identified. We used SNP to search for corresponding genes on the NCBI website and obtained 36 genes (ABCG2, ACSM2B, ALDH5A1, BCAS3, BICC1, CFAP73, CUX2, EHBP1L1, FLRT1, GCKR, GNAS, HCG22, HLA-DQB1, HNF1A, HNF4G, IGF1R, LRP2, MDC1, MLXIPL, MTX1, MXD3, NDUFAF6, NRG4, NRXN2, OR11A1, PNPLA3, PPM1K-DT, RPH3A, SESN2, SFMBT1, SLC17A1, SLC22A10, SLC2A9, SLC3A2, SLC41A1, and ZMIZ1), which may be correlated with gastric cancer and uric acid.

### 3.5 Differential gene expression in gastric cancer

Among the 36 genes included, we analyzed the differences in expression between gastric cancer and normal tissue. The results showed that there were 25 DEGs(log FC > 1, adj *p* < 0.05) compared with normal tissues. Among them, 22 genes were upregulated and three genes were downregulated in tumor tissue (Figure 4).

**Figure 4:**
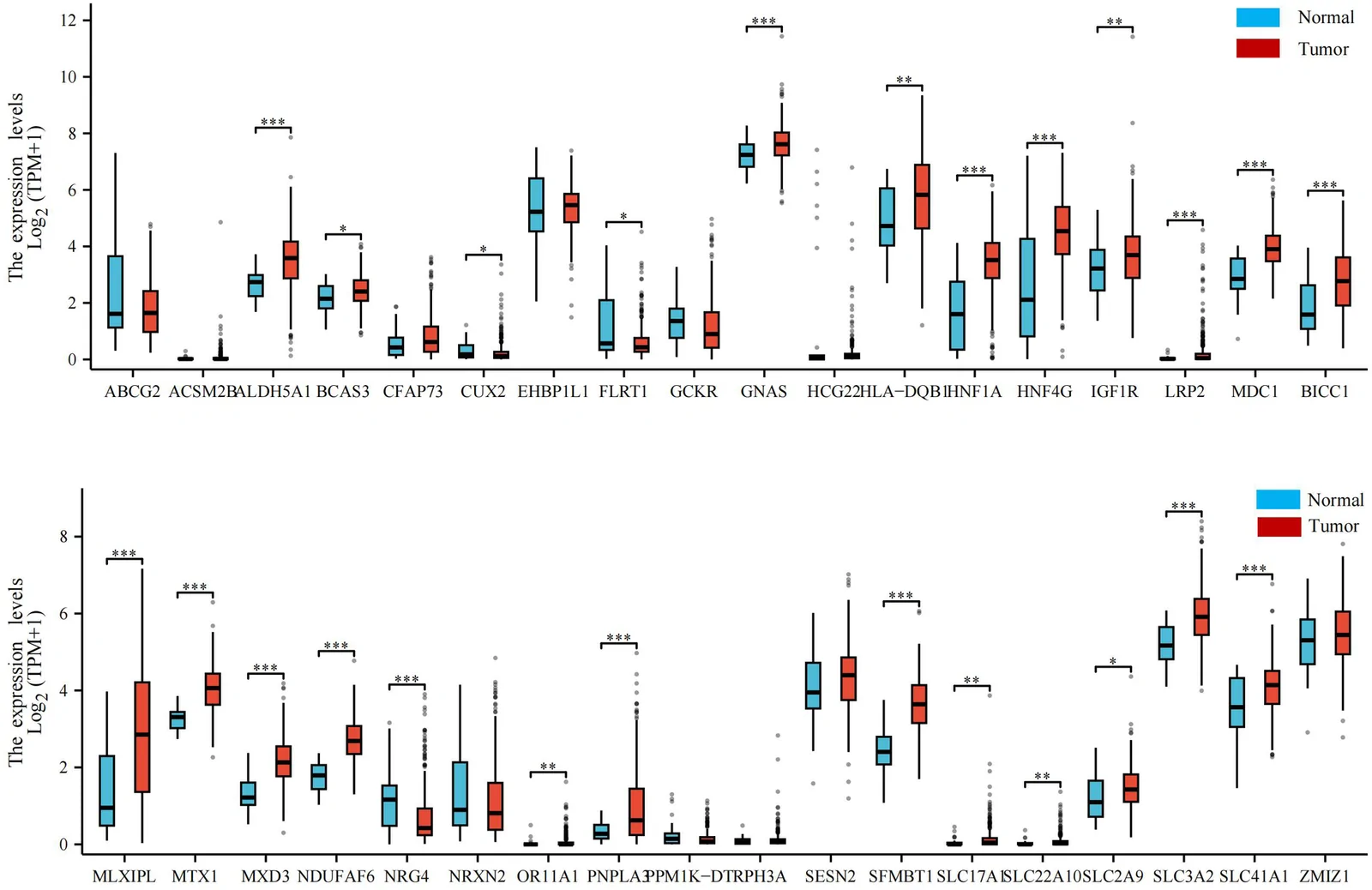
Differential expression analysis of SNP-related genes in gastric cancer. Bar plots show the expression of 36 SNP-mapped genes in gastric cancer and adjacent normal tissues.

### 3.6 Survival analysis

To further explore the potential protective role of uric acid against gastric cancer progression, we next examined the association of these SNPs with patients survival. Based on the SNP search of 36 genes, we used gastric cancer RNA-seq data from TCGA database for Kaplan–Meier survival analysis. The results showed that the expression levels of ABCG2, BICC1, CFAP73, GNAS, MXD3, and PNPLA3 were significantly correlated with the overall survival of gastric cancer patients. Among them, high expression of BICC1, ABCG2, GNAS, and PNPLA3 is a risk factor for poor prognosis in patients, whereas high expression of CFAP73 and MXD3 is a protective factor associated with better prognosis (Figure 5).

**Figure 5:**
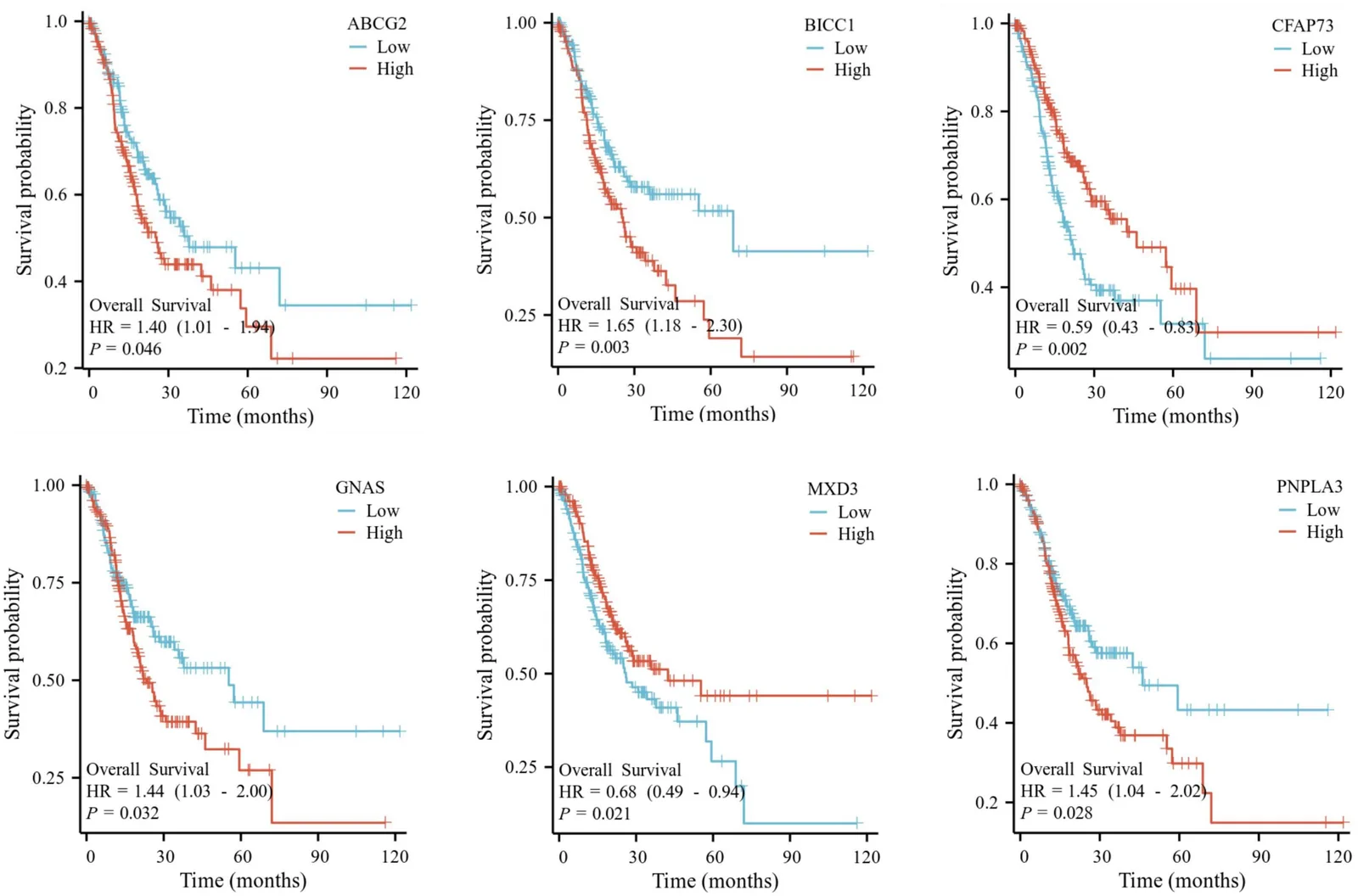
Kaplan–meier survival analysis of six SNP-related genes in gastric cancer patients. Gastric cancer patients were stratified into high- and low-expression group based on selected genes in TCGA database, and overall survival was compared between groups.

### 3.7 GO and KEGG analysis

The GO and KEGG analysis results showed that the 36 genes corresponding to SNPs were significantly associated with uric acid metabolism in gastric cancer, suggesting that they may play an important role in regulating this specific metabolic pathway in gastric cancer. At the biological process level, these genes are associated with the transport of carboxylic acids and organic anions. In terms of cellular localization, these genes were significantly concentrated in the brush border membrane and apical plasma membrane, reflecting the key pathological changes in epithelial polarity loss in gastric cancer cells. In terms of molecular function and pathways, insulin-like growth factor binding activity and the most significant activation of the mTOR signaling pathway (Figure 6).

**Figure 6:**
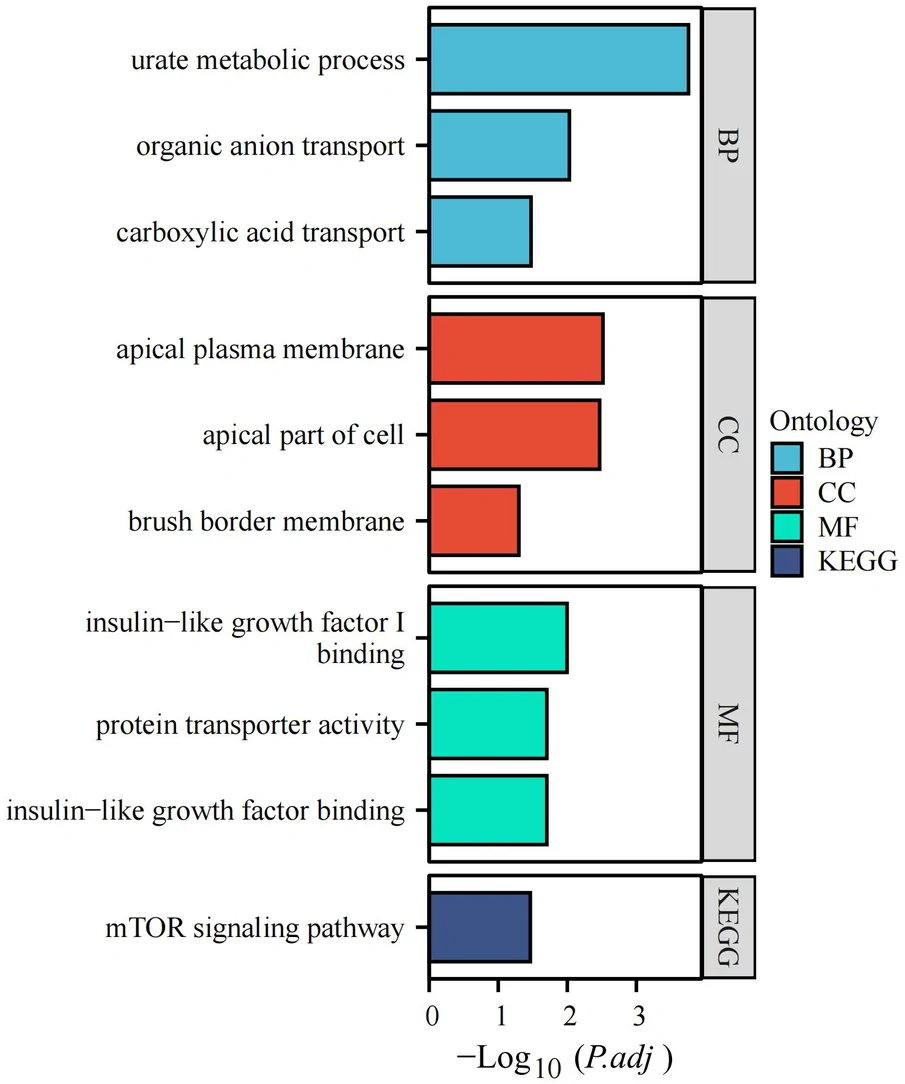
GO and KEGG enrichment analyses of 36 SNP-related genes in gastric cancer. The significant enrichment in metabolism, carboxylic acid and organic anion transport, and the mTOR signaling were noticed.

### 3.8 CCK-8 assay

After 48-hour culture in uric acid-containing medium, the CCK-8 assay results showed that the cell viability of the control group (uric acid concentration 0 µM) was approximately 100%. At a uric acid concentration of 50 µM, cell viability was approximately 90%, showing no significant difference compared with the control group. At a uric acid concentration of 100 µM, the cell viability was approximately 75%, which was significantly lower than that of the control group (*p* < 0.01). At a uric acid concentration of 200 µM, cell viability decreased to approximately 65%, which also demonstrated a significant difference compared to the control group (*p* < 0.01). These results indicated that with increasing uric acid concentration, the viability of AGS gastric cancer cells exhibited a significant dose-dependent decrease (Figure 7).

**Figure 7:**
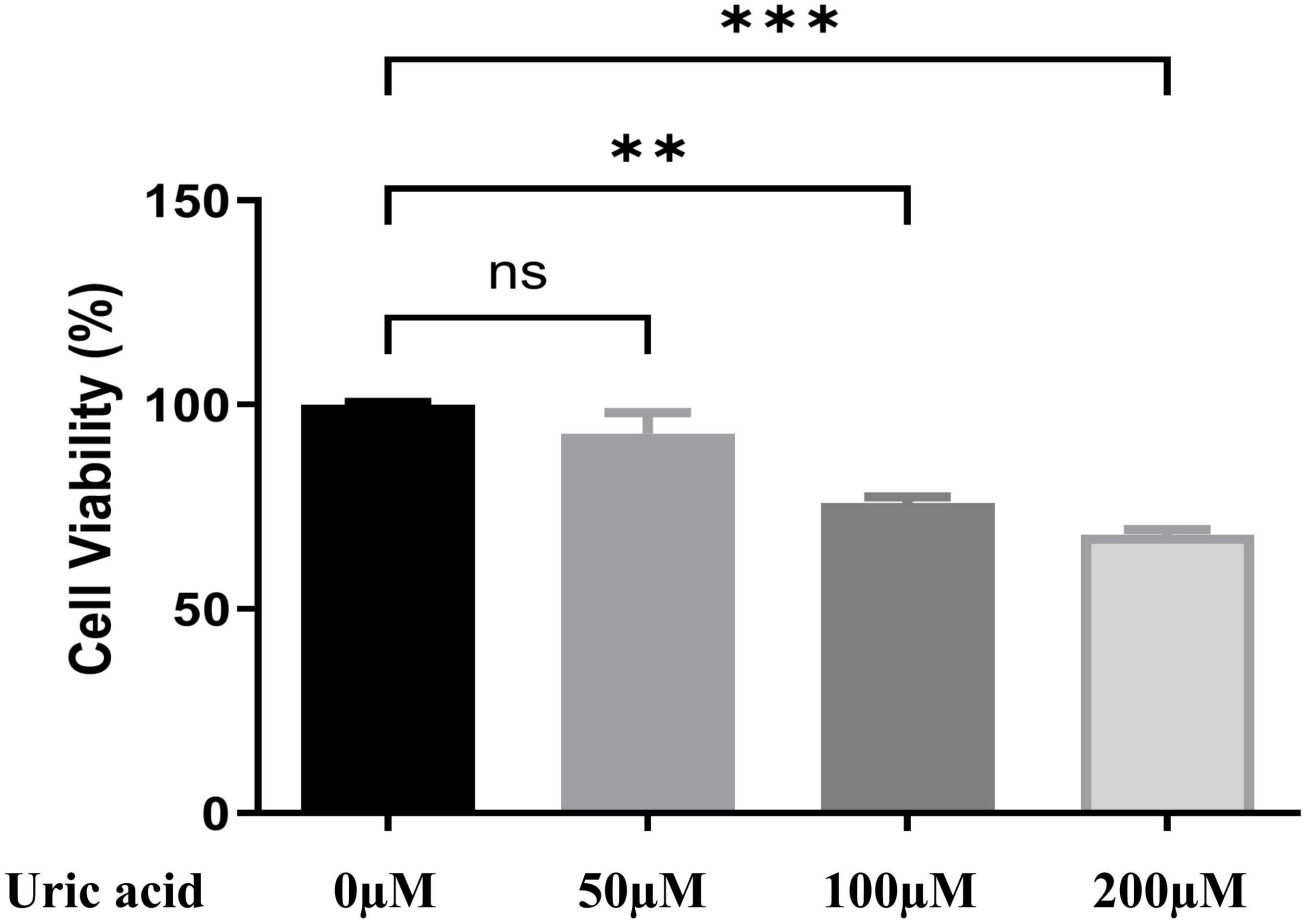
Effect of uric acid on AGS cell viability determined using CCK-8 assay. AGS cells were dosed with uric acid (0, 50, 100, and 200 μM) for 48 h and cell viability was assessed with CCK-8 kit. Data were presented as mean ± SD of three independent assays. Ns. no significance, **p < 0.01, ***p < 0.001.

### 3.9 Flow cytometry detection of apoptosis in AGS gastric cancer cells

After culturing AGS gastric cancer cells in a medium containing uric acid for 48 h, flow cytometry was performed to detect apoptosis of AGS gastric cancer cells. It was found that the proportion of the live cell population (Q1-LL) decreased slightly but continuously with increasing uric acid concentration (from 92.87% to 91.45%), while the proportion of the late-stage apoptotic/necrotic cell population (Q1-UR) showed a dose-dependent increase (from 1.83% to 2.29%), reaching its peak at 100 µM. The proportion of the early apoptotic cell population (Q1-LR) and other quadrants showed relatively small changes and no consistent trend. The results suggest that uric acid treatment (especially 100–200 µM) can induce mild but detectable late-stage apoptosis or necrosis of AGS gastric cancer cells (increased Q1-UR ratio), while the proportion of live cells decreases accordingly (Figure 8).

**Figure 8:**
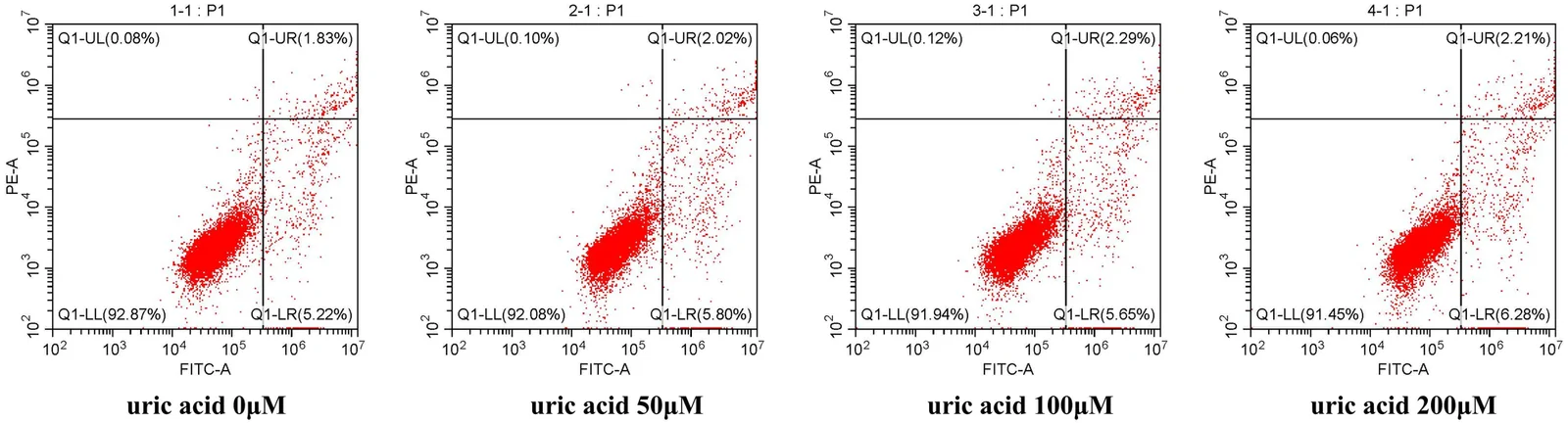
Effect of uric acid on AGS cell apoptosis. AGS cells were subjected to uric acid treatment as mentioned before. The apoptosis was detected by Annexin V-FITC/PI staining and analyzed by flow cytometry. Representative flow cytometry plots showing the proportion of viable, early apoptotic, late apoptotic, and necrotic cells in each group.

### 3.10 Flow cytometry detection of intracellular reactive oxygen species levels in AGS gastric cancer cells

After culturing in a medium containing uric acid for 48 h, flow cytometry was used to detect intracellular reactive oxygen species (ROS) levels. It was found that with an increase in uric acid treatment concentration, the proportion of high ROS cell population (V1R) increased from 32.13% to 58.09%, showing a dose-dependent increase, while the proportion of low ROS cell population (V1L) decreased from 67.87% to 41.91%. This trend indicates that uric acid treatment can effectively and dose-dependently increase ROS levels in AGS gastric cancer cells. High-concentration uric acid treatment leads to a larger proportion of cells in a high ROS state, which may be one of the potential mechanisms for inhibiting cell viability or promoting cell death (Figures 9A,B).

**Figure 9:**
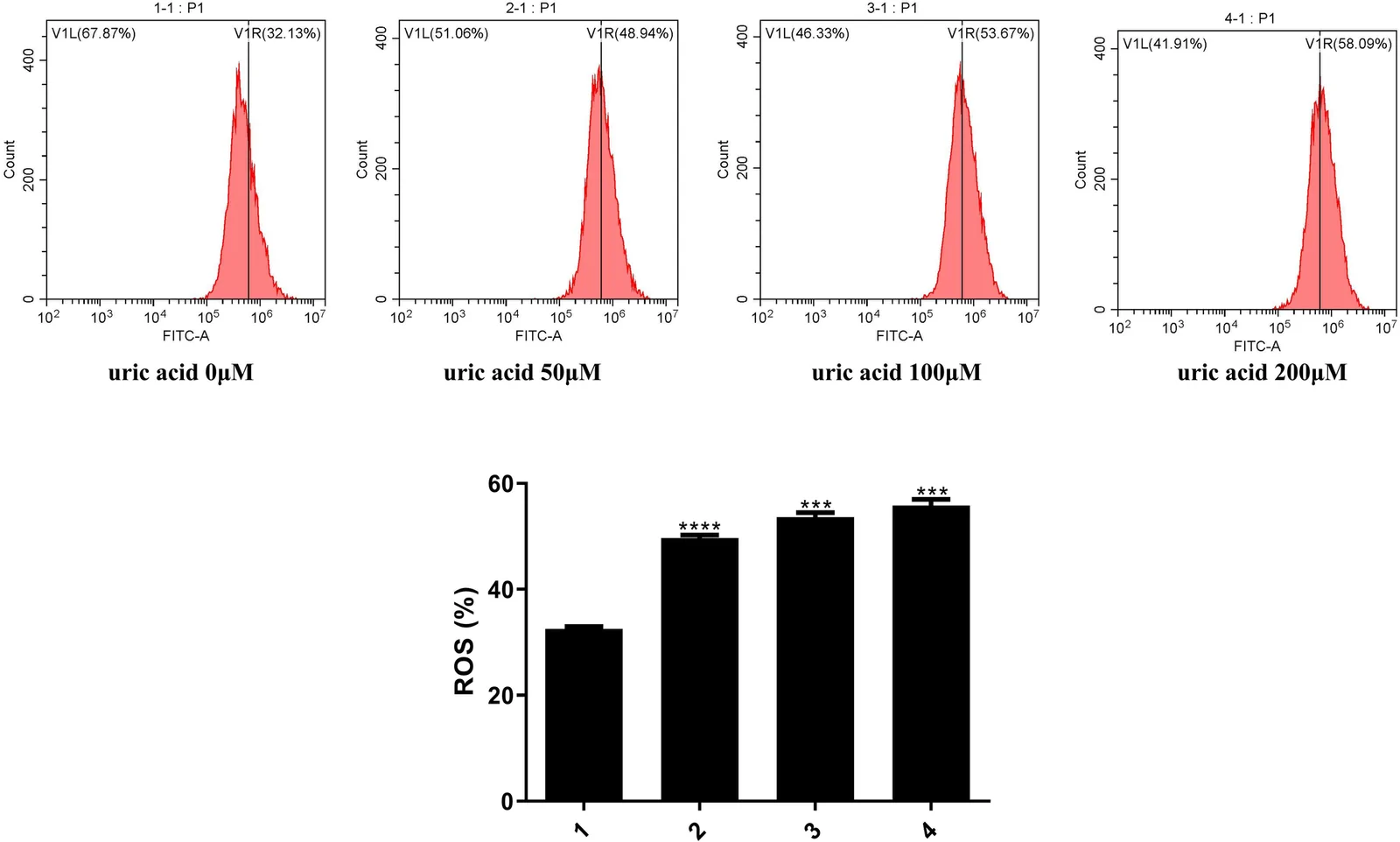
Effect of uric acid on intracellular ROS in AGS cells. After treatment with uric acid as indicated before, AGS cells were stained with DCFH-DA probe and analyzed by flow cytometry. **(A)** Representative flow cytometric plots for each treatment. **(B)** Quantitation of mean fluorescence intensity. Data were presented as mean ± SD of three independent repeats. ***p < 0.001, ****p < 0.0001.

### 3.11 Detection of levels of SOD, CAT, and GSH

Following the treatment of AGS gastric cancer cells with varying concentrations of uric acid (0 µM, 50 µM, 100 µM, 200 µM), the levels of SOD, CAT, and GSH were measured. The results showed that, compared to the control group, uric acid treatment at all tested concentrations (0 µM, 50 µM, 100 µM, 200 µM) led to a significant decrease in SOD activity (*p* < 0.05). This suggests a potential impairment of the cellular capacity to scavenge superoxide anion radicals. A dose-dependent decline in CAT activity was observed. While the decrease was not significant at 50 µM, a significant reduction in activity occurred at 100 µM and 200 µM (*p* < 0.05), indicating that higher concentrations of uric acid may compromise the cell's ability to decompose hydrogen peroxide. Intracellular GSH content also decreased with increasing uric acid concentration. The reduction was not significant at 50 µM and 100 µM, but became significant at 200 µM (*p* < 0.05). This indicates a possible depletion of reductive antioxidant reserves within cells (Figure 10).

**Figure 10:**
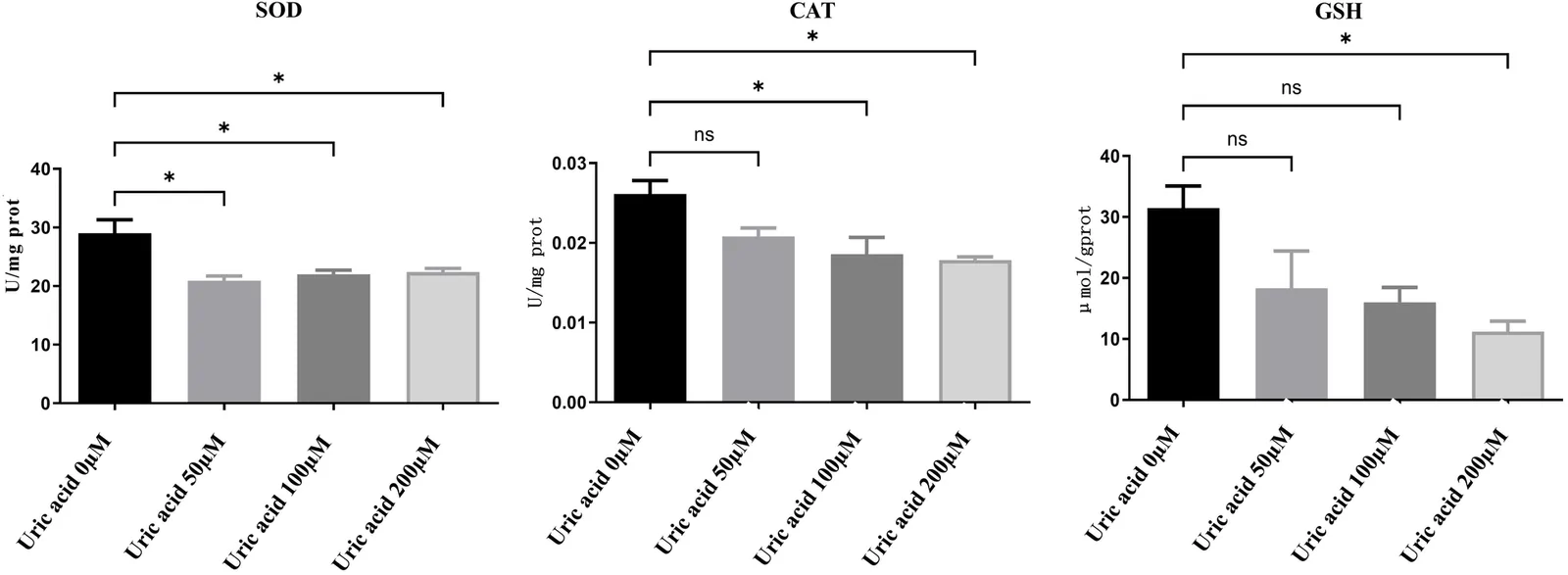
Effect of uric acid on oxidative stress markers in AGS cells. The post-treatment AGS cells were lysed and SOD, CAT and GSH were determined using commercially available kits. Data were presented as mean ± SD of three independent experiments. Ns. no significance, **p < 0.01.

### 3.12 The effect of uric acid on the activity of PI3K/akt pathway in AGS gastric cancer cells

Following the treatment of AGS gastric cancer cells with varying concentrations of uric acid (0, 50, 100, 200 µM), the protein levels of p-AKT, total AKT and PI3K were assessed via western blotting. Pathway activity was calculated as the ratio of phosphorylated AKT to total AKT, normalized to the vehicle control. The results demonstrated that compared with the control group, the relative p-AKT/total-AKT ratio progressively decreased with increasing uric acid concentration (low-dose group, 0.85; medium-dose group, 0.53; high-dose group, 0.36). These findings suggest that uric acid treatment significantly inhibits the activity of the PI3K/AKT signaling pathway in AGS gastric cancer cells. This effect manifests as a concentration-dependent reduction in the phosphorylation levels of the core AKT. These results imply that uric acid may influence the biological functions of AGS cells by suppressing the PI3K/AKT pathway (Figure 11).

**Figure 11:**
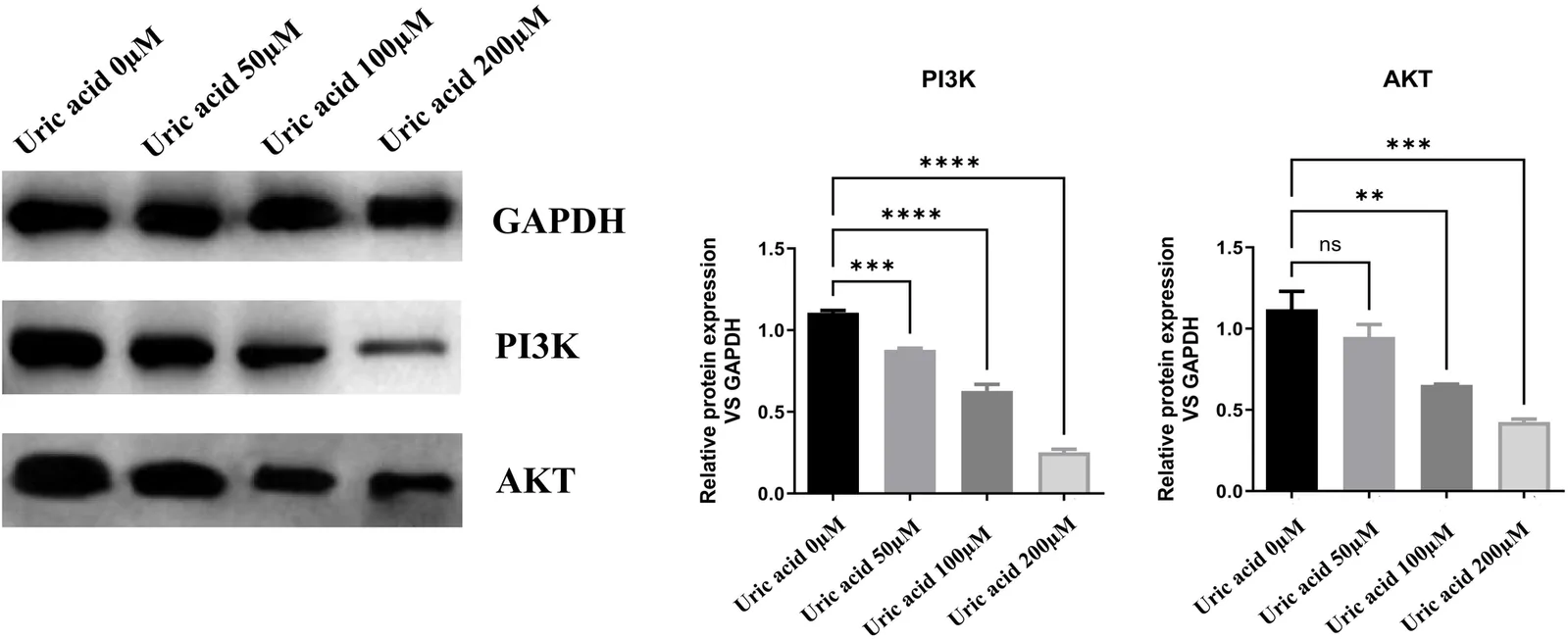
Effect of uric acid on PI3K/akt pathway in AGS cells. The protein levels of p-AKT, total ATK and PI3K in response to uric acid treatment were determined with western blot. The representative images were provided in the left panel, and quantitative results were showed in the right panel. The statistical results were acquired from three independent repeats. **p < 0.01, ***p < 0.001, ****p < 0.0001.

## 4 Discussion

This study systematically investigated the potential causal relationship between SUA levels and the risk of gastric cancer, as well as the underlying downstream molecular mechanisms, by integrating MR analysis, bioinformatic exploration, and *in vitro* functional experiments. MR findings revealed a causal association between genetically predicted higher SUA levels and a lower risk of gastric cancer development. Genes associated with SUA were found to be dysregulated in gastric cancer and to influence patient prognosis. Furthermore, *in vitro* experiments demonstrated that uric acid inhibited gastric cancer cell viability and induced cell death, potentially through mechanisms involving the induction of oxidative stress and inhibition of the PI3K/AKT signaling pathway. This may explain the observed risk-reducing effect of SUA on gastric cancer. It's noteworthy that the prognosis analysis was included in this study as supportive, secondary evidence to fully uncover the potential protective roles of uric acid beyond tumorigenesis.

The relationship between SUA levels and cancer risk has been inconsistent in previous epidemiological studies. Several meta-analyses and cohort studies have suggested a weak positive or null association between high SUA levels and overall cancer risk or the risk of specific cancers, such as colorectal cancer (2, 5, 6, 9). Regarding gastric cancer specifically, a clinical retrospective study identified high SUA as a factor for poor prognosis in patients with advanced gastric cancer (14), while a prospective cohort study suggested a potential U-shaped association between SUA and the risk of gastric cancer (16). These observational findings are susceptible to confounding factors such as renal function, metabolic syndrome, diet, and SUA alterations caused by the cancer itself or its treatment, as well as reverse causality. In contrast, MR analysis, which utilizes genetic variants fixed at conception as instrumental variables, is a more robust method for assessing causal effects. Based on large-scale population GWAS data, our study found that genetically determined SUA levels were inversely associated with the risk of gastric cancer, offering strong genetic evidence for the potential protective role of SUA. This finding differs from another MR study that reported no significant association (15), a discrepancy that may stem from differences in the GWAS datasets used, sample sizes, instrument strength, or population heterogeneity. Our results suggest that long-term, genetically determined elevation in baseline SUA levels, after accounting for numerous postnatal environmental confounders, may exert a suppressive effect on gastric carcinogenesis through its intrinsic biological properties.

To elucidate the mechanisms linking SUA to the risk of gastric cancer, we identified 36 potential mediator genes using MR analysis. These genes were significantly enriched in biological processes directly related to SUA homeostasis, such as “urate metabolic process” and “organic anion transport,” but also in processes involving “insulin-like growth factor binding” and activation of the “mTOR signaling pathway.” This suggests that SUA may regulate gastric cancer development by influencing core metabolic and signaling networks. Notably, these genes were significantly localized to cellular components like the “brush border membrane” and “apical plasma membrane,” which aligns with the pathological features of epithelial polarity loss and barrier dysfunction commonly observed in gastric carcinogenesis. This implies that SUA-related genes may be involved in maintaining the normal structure and function of gastric mucosal epithelium. More importantly, we found that the expression levels of six of these genes (ABCG2, BICC1, CFAP73, GNAS, MXD3, and PNPLA3) were significantly associated with overall survival of gastric cancer patients. This suggests a molecular link: genetic factors influence SUA levels, and SUA, along with the expression of its related genes, collectively influence gastric cancer susceptibility and progression, providing a molecular-level explanation for the complexity of the SUA-gastric cancer relationship.

It has been noted that uric acid plays a “dual role” in cancer, acting as a potent antioxidant at physiological concentrations and potentially as a pro-oxidant at high intracellular concentrations (4). Our cell experiments showed that exogenous uric acid treatment inhibited the viability of AGS gastric cancer cells and induced late apoptosis/necrosis, directly supporting its anticancer activity at the cellular level. This pro-apoptotic effect was accompanied by a significant increase in intracellular reactive oxygen species (ROS) levels, decreased activities of superoxide dismutase and catalase, and reduced glutathione content. This indicates that uric acid may primarily exert a “pro-oxidative stress” effect in gastric cancer cells, triggering apoptotic signals by disrupting intracellular redox homeostasis.

Regarding the specific pathway-related mechanisms, we found that uric acid significantly suppressed the expression of key proteins in the PI3K/AKT signaling pathway. The PI3K/AKT pathway is a central regulator of cell survival, proliferation, and metabolism, and its hyperactivation is closely associated with the progression of various cancers including gastric cancer. The inhibition of this pathway by uric acid, synergizing with induced oxidative stress, may be a key molecular event underlying its suppression of gastric cancer cell growth. However, this result appears to contradict our bioinformatic enrichment finding of “mTOR signaling pathway” activation (PI3K/AKT is a major upstream activator of mTOR). This discrepancy may arise from differences between the complex *in vivo* regulation and acute pharmacological effects of direct *in vitro* uric acid treatment. This may also suggest that the long-term genetic influence of SUA-related genes and the acute effect of uric acid molecules involve distinct mechanistic emphases, warranting further in-depth experimental exploration.

In summary, we proposed a preliminary model: a genetically elevated baseline SUA level may confer a protective effect during the initial stages of gastric carcinogenesis, potentially through its antioxidant properties or by regulating a set of genes involved in urate metabolism, transport, and epithelial polarity maintenance. This is consistent with the MR findings. However, in established or advanced gastric cancer, the role of uric acid may shift towards a pro-oxidative and pro-inflammatory direction, correlating with a poorer prognosis (14), and potentially activating pro-survival pathways, such as mTOR, as an adaptive response to a hostile microenvironment. Our *in vitro* experiments, simulating the direct effect of relatively high uric acid concentrations on already transformed cells, revealed its potential therapeutic value in inhibiting tumor cell growth by inducing oxidative stress and suppressing the PI3K/AKT pathway.

This study had several limitations. The MR analysis primarily indicates a long-term causal trend but cannot reveal dynamic changes or nonlinear relationships (e.g., U-shaped associations) (5, 16), and only the IVW method showed statistical significance in the MR analysis. Heterogeneity was present, but leave-one-out and other sensitivity analysis supported the robustness of the causal estimate in this study. Second, the exposure and outcome GWAS was performed in different ancestral populations, which may partially violate the assumptions of two-sample Mendelian randomization. The 1,000 Genomes European reference panel used for LD clumping may not fully reflect the LD structure of the East Asian population as well. Therefore, the results should be interpreted cautiously in view of these potential disturbances, and future studies using ancestry-matched GWAS datasets are warranted to further validate the causal relation between SUA and gastric cancer. Cell experiments were conducted in a single gastric cancer cell line, and further validation in more gastric cancer cell lines and animal models is necessary. The specific functions of the candidate genes and how they mediate the effects of SUA require further investigation through functional gain- and loss-of-function experiments. Finally, the prognostic analysis based on retrospective cohort data should be interpreted as supportive evidence rather than direct proof of a protective effect of uric acid against gastric cancer risk.

## 5 Conclusion

This study provides, for the first time, genetic evidence from MR analysis that supports an inverse causal relationship between SUA and the risk of gastric cancer. Through multi-omics analysis and functional experiments, we revealed the potential molecular and cellular mechanisms underlying this association. These findings not only deepen the understanding of the complex role of uric acid in gastric cancer but also offer novel insights for exploring gastric cancer prevention and adjuvant treatment strategies targeting urate metabolic pathways.

## Data Availability

The original contributions presented in the study are included in the article/Supplementary Material, further inquiries can be directed to the corresponding author.
